# Comparative Study on Thermal Behaviour, Tool Wear and Surface Roughness in Milling EN8 Steel for Sustainable Machining

**DOI:** 10.3390/ma19050975

**Published:** 2026-03-03

**Authors:** Thenarasu Mohanavelu, Narassima Madhavarao Seshadri, Sreeranjani Vijayakumar, Sumesh Arangot, Jana Petru, Saravanamurugan Sundaram

**Affiliations:** 1 Department of Mechanical Engineering, Amrita School of Engineering, Amrita Vishwa Vidyapeetham, Coimbatore 641112, India; cb.en.u4mee23041@cb.students.amrita.edu (S.V.); a_sumesh@cb.amrita.edu (S.A.); s_saravana@cb.amrita.edu (S.S.); 2 Great Lakes Institute of Management, Chennai 603102, India; narassima.s@greatlakes.edu.in; 3 Department of Machining, Assembly and Engineering Metrology, Faculty of Mechanical Engineering, VSB-Technical University of Ostrava, 70800 Ostrava, Czech Republic

**Keywords:** sustainable machining, response surface methodology (RSM), grey relational analysis (GRA), tool wear and surface roughness, thermal imaging

## Abstract

Dry machining of medium-carbon steels plays an important role in sustainable manufacturing; however, high tool wear and thermal instability pose challenges. The study aims to evaluate the kinematic–tribological performance of EN8 steel during dry milling and compare up-milling and down-milling to trade-off tool life and surface finish. The experiments were conducted using a central composite design (CCD) as part of response surface methodology (RSM), with 36 runs to evaluate interactions among spindle speed, feed rate, and depth of cut. Down-milling outperformed up-milling, achieving 12.4% less tool wear, 45.9% better surface finish, and a 47 °C lower peak temperature from cutting. The above benefits are attributed to the unique kinematics of chip formation during down-milling, which offers lower friction at entry and better heat dissipation, contrasting with the high-friction ploughing phase of up-milling. Grey relational analysis (GRA) found that down-milling with a mid-range cutting speed (22.31 m/min) and a low feed rate (25 mm/min) provided a multi-objective optimum. The findings support the existence of a kinematic–tribological coupling, providing a solid single approach to optimising the dry machining of harder materials.

## 1. Introduction

Medium-carbon EN8 steel offers a good combination of strength, toughness and wear resistance, meaning that it is often selected for machining load-bearing components such as shafts, gears, bolts and pins. When EN8 steel is heat-treated, it can be made to be harder and to resist more wear than it could otherwise. While carbon levels are an essential contributor to the increased hardness and wear resistance of this material, it also causes work hardening during machining, leading to increased tool wear and lower surface quality on milled components, due to the heat generated in the cutting zone and the material’s low thermal conductivity [1].

In milling, a rotating multi-edge cutter makes several passes to remove material. The two main types of milling are up-milling and down-milling. The cutter rotates in the opposite direction to the feed direction (up or conventional milling), resulting in an increasing chip thickness from 0 at first to its maximum. Before cutting begins, there is considerable rubbing and ploughing, causing excessive friction, heat, and force spikes due to work-hardening of the surface, leading to increased abrasive wear and limited surface finishes. In contrast to down-milling (climb milling), where the cutter rotates with the feed, down-milling engages the cutting tool by eliminating the thickness of the chips at the start and reducing it to nothing while producing less friction, better chip removal, and improved force transfer, which in turn leads to enhanced surface finish production and reduced tool wear [2]. Cemented carbide tools are the preferred option for machining EN8 steel due to their high hardness and resistance to thermal and abrasive wear. Carbides also retain cutting ability at higher temperatures than high-speed steel, making them ideal for dry (no coolant applied) milling operations. Additionally, the toughness of carbide tooling helps maintain cutting edges, resulting in consistently longer tool life and improved part quality [3].

Response surface methodology (RSM) has developed statistical modelling techniques to allow multiple outputs from one or more inputs, and it has used GRA as an integrated measure of performance across all input/output pairs. In a machining environment, RSM assists in identifying relationships among various cutting conditions, such as spindle rotation speed, feed rate, and cutting depth, and contrasting the performance of machining processes using measures of tool wear, surface roughness, and cutting temperature (i.e., surface corrosion and/or melting). Additionally, RSM can develop predictive models from a minimal number of experiments, enabling the identification of optimised machining settings to improve overall performance [4]. Analysis of variance (ANOVA) serves as an adjunct to RSM, as it quantifies the influence of each input on each output; thus, RSM can help validate ANOVA results and identify which input variables play the most significant role in a specific production [5]. Both tool wear and surface roughness are key indicators of a milling operation’s overall efficiency and product quality. Most previous research, however, has looked at these variables separately or has focused solely on one orientation of tool movement. As a result, there is a lack of a comprehensive comparison of the differences between up-milling and down-milling for EN8 steel, evaluated under the same conditions using all three major response factors.

Although EN8 steel is a standard grade used in factories of all types, no studies provide a detailed analysis of the combined effects of thermal, tribological, and surface properties on the machining performance of EN8 steel when dry. Therefore, to fill this knowledge gap and strengthen scientific understanding of how process parameters influence tool life (i.e., milling process optimisation), this study proposes the following research objectives:

RQ1. What are the differences between up-milling and down-milling during the dry machining of EN8 steel?
RQ2. How do non-linear interaction effects between feed rate, cutting speed, and depth of cut impact the thermal and tribological performance of uncoated carbide cutting tools?
RQ3. Is it possible to address the trade-off between tool life and surface quality?

The purpose of this research was to compare and assess the effect of tool wear (wear), surface finish quality (roughness), and cutting temperature when machining EN8 steel with two different milling techniques: up-milling and down-milling. The experiments were performed on a semi-automatic vertical milling machine under dry-cutting conditions, using a 10 mm-diameter uncoated solid carbide endmill. Throughout the 36 experiments, the spindle revolutions per minute (RPM), feed rate, and depth of cut were varied using experimental RSM.

Optical microscopic analysis of tool wear was performed after each trial, and surface finish was measured using profilometry. A thermal imaging camera was used to measure peak cutting temperature using infrared radiation. Statistical methods were employed to develop distinct models for the response variables and to conduct an ANOVA to identify significant factors and interactions influencing tool life and surface finish.

The study systematically compared the impact of up-milling and down-milling EN8 steel through dry machining by use of response surface methodology (RSM) to develop cutting parameters, then performed an analysis of variance (ANOVA) on those parameters. Additionally, we included a grey relational analysis (GRA) to express relationships between the multiple output variables from both milling methods and to allow for a full understanding of the differences between each method and under each experimental condition. The research will be used to optimise the advantages of keeping tool life, surface finish, and machining temperature under control. The structure of this paper is as follows: Introduction and literature review discussed in this section followed by Section 2. Methodology, Section 3. Results, Section 4. Discussion, Section 5. Theoretical and Practical Implications, and Section 6. Conclusions and Future Work.

### 1.1. Thermal and Wear Behaviour in Milling of EN8 Steel

Numerous studies have shown that cutting temperatures affects tool wear and the overall quality of workpieces. Most of the heat generated during machining originates from the friction between the tool and workpiece (primary heating), followed by the friction between the tool and chip (secondary heating), and finally, the heat produced by the tool’s clearance surface (tertiary heating). In fact, the results indicate that approximately 80–90% of the heat generated by the machining process is absorbed by the chip, with only 10–15% affecting the cutting tool or workpiece [6], thereby influencing tool wear, surface roughness, and dimensional accuracy. Increasing cutting speeds also increases cutting temperature, increasing the risk of thermal damage to the tool, as well as oxidation and diffusion wear on its surface. The maximum cutting temperature depends on the milling parameters; up-milling produces lower chip surface temperatures than down-milling due to differences in chip formation and tool positioning [7].

Experimental results on cutting conditions have shown that, at higher temperatures, the machined surface hardness increases, leading to difficulties in machining and higher surface roughness. Dry milling exhibits both abrasive and adhesive tool wear modes. The effects of oxidative wear have also been identified, to a lesser degree, as impacting surface roughness and tool wear resistance [8]. The comparison between wear on tooling and the surface quality produced by different milling processes, using these results, has been completed. It has also been noted that both the helix angle and feed rate contribute to surface roughness, as reported in previous studies [9]. The simulation techniques used to model surface topography provide a basis for further studies of differences in surface roughness between up-milling and down-milling [10]. The results of this study indicate that, when comparing surface roughness and chip formation characteristics for both up-milling and down-milling, down-milling produces a finer surface finish due to the distribution of compressive stresses and the minimisation of built-up edge formation [11]. The addition of alloying elements affects surface finishing by altering the microstructure and forming brittle intermetallics. Increased cutting speeds and temperatures lead to lower surface quality [12]. This study evaluates cutting temperature and surface quality by analysing residual stresses in high-speed milling. It summarises the observation that elevated temperatures and cutting forces generate tensile residual stresses, degrading surface integrity, whereas compressive residual stresses improve it [13]. The relationship between the tool material and cutting speed on these two parameters (tool wear rate and surface roughness) will be shown to be as follows: down-milling yields lower wear rates due to lower cutting forces [14]. The relationship between tool wear and heat buildup, while also allowing effective determination of wear rate in terms of overall tool wear and the relationship between tool wear and heat (and tool wear on a tool’s life through the finished product), is the primary subject of this research on the thermal–mechanical interactions that result in tool wear and deformation from high-speed machining [15]. Cutting speed is found to play a major role in cutting force, flank temperature, and tool wear in milling operations. The temperature evolution and plastic deformation during the milling process demonstrate that cutting direction influences the progression of tool wear [16]. The heat formed by the tool as it cuts through the material (both up-milling and down-milling) can be utilized to predict wear on tools and quality of machined parts [17].

### 1.2. Influence of Milling Strategy and Process Parameters

Down-milling enhances tool wear performance and efficiency over up-milling processes, as there is an increased amount of heat generated from the cutting process when compared with up-milling. According to the conducted experiments, a lower cutting force has been recorded, as well as reduced temperatures at the interface of the tooling and workpiece, when using down-milling versus up-milling [18,19]. Furthermore, the use of more sophisticated cooling techniques, such as liquid nitrogen (LN2) or minimum quantity lubrication (MQL), has improved the quality and performance characteristics of down-milled parts compared with similar up-milled parts [20]. Studies have shown a strong relationship between the feed rates and cutting speeds of a cutting machine and their impact on the amount of tool wear, burr formation and surface finish produced during machining. This further demonstrates that the feed rate and cutting speed are directly related to the tool’s tool life and surface finish [21]. Tool wear variation affects both surface integrity and the fatigue life of machined parts [22]. Analysis of comparisons between down-milling and up-milling cutting forces indicates that less cutting force and less heat will be generated during down-milling operations (therefore longer tool life and better surface finish) [23]. Chip removal occurs in the downward direction of down-milling, which assists in removing chips and prevents tool adhesion/transfers the tool cuts through the workpiece as compared with up-milling; therefore, there is less stress on the workpiece as well as reduced tool wear [24]. Because of the nature of up-milling, the tool cuts downward into the work, generating heat, requiring greater cutting force, and creating abrasive forces that result in poor surface quality and increased tool wear [25]. Heat generated during up-milling not only increases tool wear but also increases the roughness of the machined surface. Using the down-milling method, the downward direction and the chip flow can relieve such stressors on the tool, providing a much smoother surface finish and longer tool life. According to the study, it is also best to optimise cutting speed and cooling strategies to achieve maximum benefits [26].

In the machining of composite materials, researchers used multiple optimisation techniques to handle complex process interactions [27]. A study on the machining of LM25 aluminium reinforced with vanadium carbide compared different modelling approaches, including analytical models, deep neural networks (DNNs) and GRA integrated with RSM [28]. The analysis showed that DNN was able to strike a much better balance between the material removal rate (MRR) vs. surface quality (SQ) and energy efficiency (EE) blend. In addition to DNN/GRA, comparison studies of the electric-discharge coating process using RSM, artificial neural network (ANN), and the adaptive neuro-fuzzy inference system (ANFIS) were conducted for both thickness and hardness predictions. ANFIS produced better results than either RSM or ANN; most notably, ANFIS predictions provided greater accuracy than either RSM or ANN under conditions of less compaction loads [29]. The construction industry has also benefited from the use of these optimisation tools. Several studies on concrete containing waste glass powder applied ANOVA for statistical testing and RSM for modelling the effects of partial cement replacement [30]. These methods have helped in the effective understanding and optimisation of mixture properties. In biodiesel production, research on the conversion of yellow oleander seed oil used both RSM and ANN for prediction. Both models achieved high levels of accuracy, with RSM reaching an R^2^ of 99.98% and ANN achieving 99.94% [31]. Studies across various materials have identified the principal input variables that exert the most significant influence on machining parameter optimisation [32].

In the case of aluminium alloys, when using a combination of the Taguchi method with ANOVA, feed rate was found to have the most significant effect on surface roughness, followed by cutting speed and depth of cut [33]. Studies related to controlling cutting temperature, cutting speed and depth of cut were also identified as key contributors to the generation of cutting temperature [34]. Optimising these parameters resulted in a significant reduction in cutting temperature by up to 27% [35,36]. Literature reviews have provided researchers with a comprehensive understanding of tool wear in both up-milling and down-milling. The primary modes of wear are identified as abrasive, adhesive, and oxidative, with cutting speed, tool coating, and heat generation influencing tool life and surface finish quality [37,38].

### 1.3. Research Gap and Novelty

Although considerable research has been conducted on tool wear and surface quality during milling, little research has examined the specific relationship between the tool wear mechanism and cutting direction in the machining of harder materials, such as EN8 steel [39,40]. Existing investigation on EN series steels highlights wear behaviour but does not explicitly correlate it with milling direction [41], revealing a clear research gap. General studies on machining parameters further confirm their strong influence on tool life [42,43]. Some key outcomes are presented in Table 1.

This study aims to address that gap by focusing on how up-milling and down-milling influence tool wear, surface roughness and cutting temperature during the machining of EN8 steel. The objective is to compare the performance of both milling strategies under identical test conditions and to understand how cutting parameters affect wear, thermal behaviour and surface finish. To achieve this, the study was limited to the dry milling of EN8 steel using an uncoated solid carbide cutter on a semi-automated milling machine. No coolant or advanced monitoring systems were used. The work relied entirely on physical experiments, without any simulation or modelling. RSM and ANOVA were used to analyse the relationship between process inputs and measured outputs.

The key research gap identified is that previous studies typically focused on either up-milling or down-milling, or on a single response, such as tool wear or surface finish. No prior work has compared both milling methods on EN8 steel in the same set of trials while examining tool wear, surface roughness and cutting temperature together. This work is novel because it applies a single, unified set of 36 experiments and standard statistical approaches (RSM and ANOVA) to examine how speed, feed, depth of cut, and milling direction affect all three machining responses. No earlier research has provided predictive models covering wear, finish and heat under matched conditions for both up- and down-milling. From a practical standpoint, the results recommend using down-milling with a moderate spindle speed, a low feed rate, and a 0.5 mm depth of cut to reduce tool wear by about 25%, improve the surface finish by roughly 30%, and keep the temperature increase within 7 °C. The regression models developed through RSM and validated by ANOVA can also be incorporated into computer numerical control (CNC) machines for the real-time monitoring of tool wear and temperature, potentially reducing tool change frequency and overall machining costs.

Future studies can build on this work by incorporating cutting force and vibration data to explore the role of machine dynamics in tool wear. Additional research can also focus on the use of coated tools or cooling techniques, such as minimum quantity lubrication (MQL) or cryogenic cooling, to further minimise heat and wear. Applying machine learning to live wear prediction and process control, in combination with the RSM–ANOVA framework used here, may provide even deeper insights into optimising the machining of medium-carbon steels such as EN8.

## 2. Methodology

**Figure 1:**
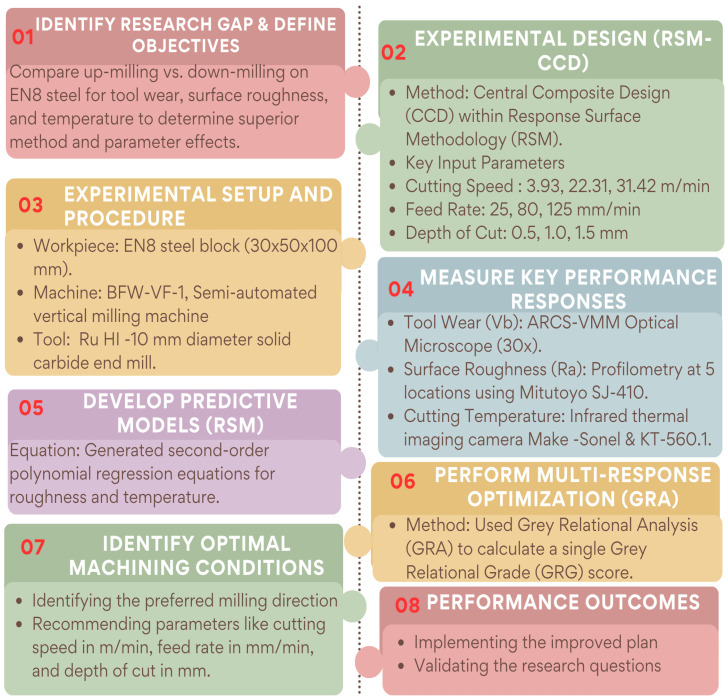
Methodology framework.

The objective of this research study is to determine how milling direction and cutting parameter selection affects tool wear and surface finish during the dry machining of EN8 steel. The experimental plan for the study was developed using a central composite design (CCD) and RSM. The CCD enabled the development of an efficient experimental method and generated predictive models from fewer experimental runs, which were then used to determine the optimal cutting conditions. The main process parameters for the study were cutting speed, feed rate, and depth of cut, which were treated as categorical variables for both down-milling and up-milling. A flowchart (shown as Figure 1) has been created to give a visual illustration of the step-by-step approach taken in this research and the framework for obtaining accurate, repeatable and applicable results to be used in the manufacturing industry. Below the flow chart is an explanation of the types of materials (workpieces) and tools used throughout the project, the machine configurations employed to complete this project (including measuring instruments), the measurements obtained during the course of the study, the experimental parameters selected to be examined and, finally, the method of analysis applied to the data received from the experiment.

### 2.1. Workpiece and Cutting Tool

The study examined EN8 alloy, a medium-carbon steel with very high impact toughness and high wear resistance. These characteristics make it ideal for mechanical engineering applications, such as shafts, gears, and bolts. The specimen was prepared as a rectangular block measuring 30 mm × 50 mm × 100 mm to allow for secure clamping and cutting in the mill apparatus. As it has a hardness of 28 on the Rockwell hardness scale (HRC), EN8 can be machined dry without requiring any additional heat treatment. To ensure an accurate test, an initial facing operation was performed to create a uniform surface before measuring tool wear and roughness. The chemical composition (by weight) of EN8 Alloy is as follows: Carbon 0.36%, manganese 0.6%, silicon 0.05%, phosphorus 0.015%, and sulphur 0.015%. The reported composition is based on standard EN8 (080M40) specifications as defined under BS 970. All milling operations were carried out using uncoated standard solid carbide end mills with a high level of hardness and the ability to maintain cutting performance at elevated temperatures generated during dry cutting. All tools were four-flute solid carbide end mills with a diameter of 10 mm and a helix angle of 30°, designed with a corner-radius end geometry for improved stability during machining. The cutters, manufactured by Ru HI, featured a corner radius of 0.5 mm. The tool specifications included a flute length of 22 mm, a shank diameter of 10 mm, and an overall length of 72 mm. To provide a side-by-side, isolated comparison of the wear patterns between the two tools used in this study, one tool was designated solely for up-milling, and the second for down-milling. As the tools lacked coating, direct observation of the interaction between the tool and the workpiece was possible. Prior to use, the cutting edge of each cutter was checked for consistency of geometry and sharpness to determine surface quality and wear evaluations during testing.

### 2.2. Machine Tool and Setup

**Figure 2:**
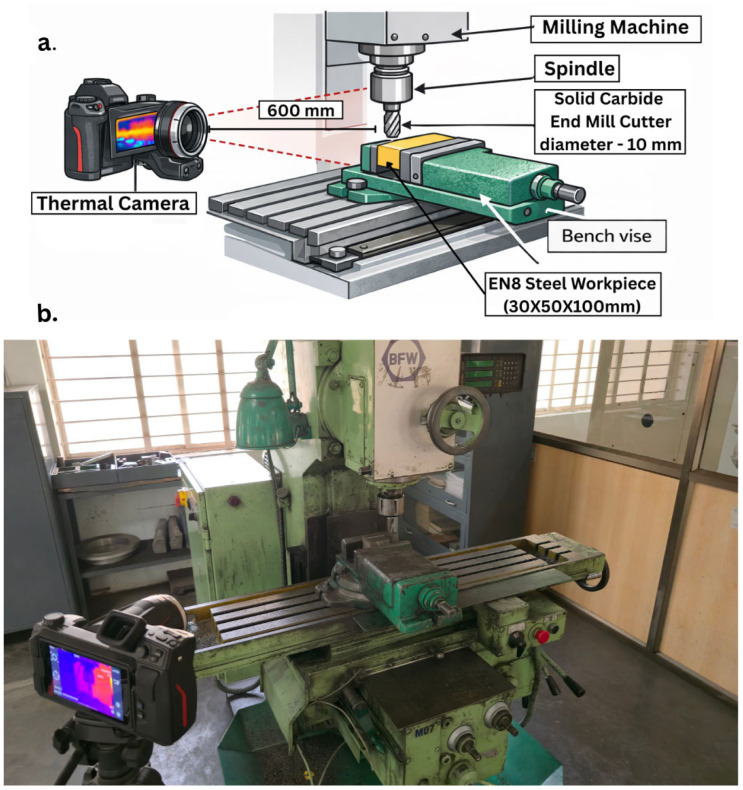
Experimental setup. (**a**) Schematic representation and (**b**) laboratory arrangement.

Milling experiments were performed using a semi-automated universal vertical milling machine (BFW VF-1) manufactured by Bharat Fritz Werner Limited (Bengaluru, India), equipped with an integrated automatic feeding system to maintain constant feeding rates and reduce human error. The machine’s clamping area is 1000 mm × 230 mm with a 4-horsepower (H.P.) spindle motor and a 0.75 H.P. feed motor. It can accommodate spindle speeds from 45 to 2000 RPM and feed speeds from 16 to 800 mm/min. The overall arrangement of the machine tool system including the workpiece hold-down, cutter tool, thermal monitoring systems, etc. are shown in Figure 2.

The spindle will rotate in the clockwise direction while the feed direction changes on each pass. The down-milling process runs the table from left to right on the front view, and the cutter and workpiece move together, while the up-milling process will run the table from right to left with the cutter turning against the feeder direction. The use of carbide end mills mounted in a standard tool holder result in a very rigid and low runout and/or deflection of the cutter during use.

An EN8 steel workpiece was securely fastened to a vice attached to the milling table to prevent any vibration or movement during machining processes. During machining, an IR thermal imaging camera was placed at a stationary position in front of the milling machine (i.e., facing the interface between the tool and workpiece) to provide continuous temperature monitoring during the testing period.

### 2.3. Selection of Parameters and Experimental Design

The spindle speeds, feed rates and depth of cut chosen for this study came from a review of previous studies and information obtained from a variety of sources. These parameters were chosen to obtain measurable differences in tool wear and surface finish, while operating safely within the thermal and mechanical limits of the machine and the limitations of uncoated tools.

The cutting speeds were chosen from 3.93 m/min to 31.42 m/min, which is appropriate for cutting medium-carbon steels. The feed rates selected, 25 to 80 to 125 mm/min, included both fine finish and aggressive removal applications. The depths of cut selected were 0.5, 1.0, and 1.5 mm to evaluate the impact of thermal loads on light and moderate engagement. The machining parameters and their corresponding levels are listed in Table 2.

To evaluate the effects of these parameters, a CCD was developed using RSM. This method provides an efficient means of designing an experiment to estimate linear, quadratic, and interaction effects for factors at three levels with the minimum number of trials. The experimental plan consisted of 40 trials, providing an equal data set for validating the regression models and ANOVA.

### 2.4. Response Measurement and Data Analysis

**Figure 3:**
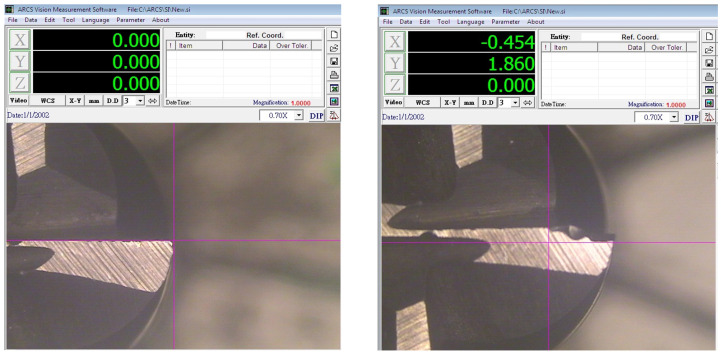
Microscopic image for tool wear measurement of the up-milling tool: initial tool wear of tip-1 (**left**) and 2nd trial tool wear of tip-1 (**right**).

Experimental runs were conducted in a simulated environment without any coolant for dry milling operations. To monitor the progress of tool life, flank wear (Vb) was measured after every three consecutive passes using an HO-TMM-01 optical microscope combined with ARCS Vision Measurement software (ARCS Precision Technology, Taichung, Taiwan, China). This microscope has a total magnification of 30× and a resolution of 0.003 mm, providing accurate measurement of tool wear. Each time the machine stopped between passes, the tool was allowed to cool down to ambient temperature, thus avoiding subsequent cutting temperatures due to thermal accumulation. Representative tool wear images captured using the vision measurement system are shown in Figure 3.

**Figure 4:**
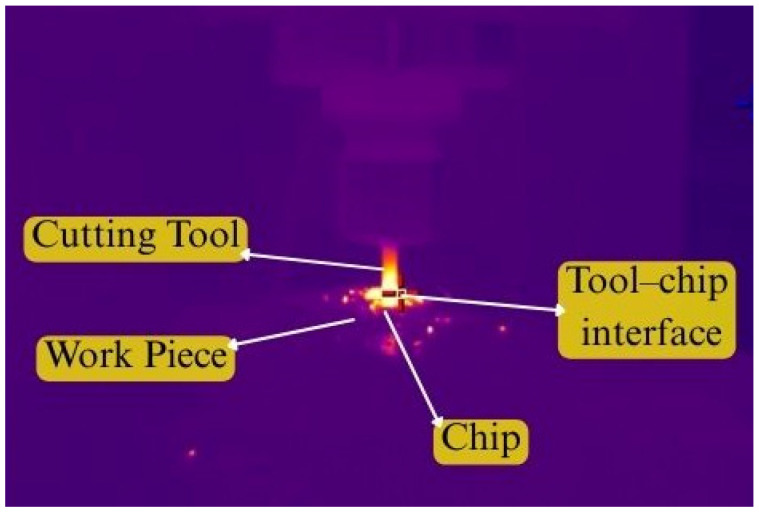
Thermal imaging during EN8 milling.

Cutting temperatures were continuously measured during each machining pass using an infrared thermal camera. A Sonel KT-560 infrared thermal imaging camera (Świdnica, Poland) was used for this purpose. The camera was positioned at a measured distance of 600 mm from the cutting zone to ensure safe and uninterrupted monitoring of the machining process. The emissivity value was set to 0.75 to account for the radiative characteristics of the tool–chip interaction zone and the freshly formed chip surface. The camera has a thermal sensitivity of 45 mK, and measurements were conducted under an ambient relative humidity of approximately 70% RH. The infrared thermal camera had a thermal sensitivity of 40 mK and an accuracy of ±2 °C or ±2%. It is important to note that, due to chip obstruction and tool rotation, direct measurement of the peak temperature at the cutting edge is difficult. Therefore, the recorded temperature corresponds to the tool–chip interaction region rather than the absolute maximum tool-tip temperature as shown in Figure 4.

**Figure 5:**
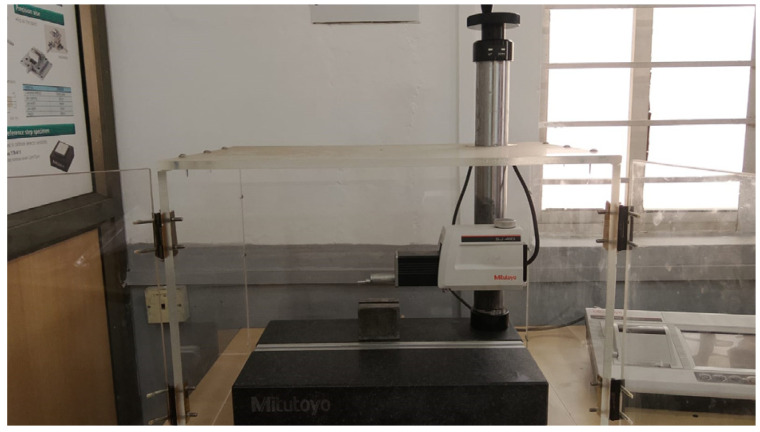
Surface roughness tester.

The surface roughness (Ra) was measured after all the machining passes using a Mitutoyo (SJ-410) column-type surface roughness tester (Świdnica, Poland), as shown in Figure 5. Surface roughness measurements were taken at 5 locations along the length of the machined piece, with the average representing the trial’s surface roughness.

Statistical data analysis was performed. The relationship between input parameters and responses were modelled using second-order polynomial regression equations derived from RSM. To validate these models, ANOVA was conducted to examine statistically significant differences (*p*-values < 0.05). In addition, GRA was used to rank machining conditions using multi-objective optimisation (i.e., minimising flank wear, surface roughness, and temperature), producing a single performance index to determine the optimal machining condition settings.

## 3. Results

This section presents the experimental results obtained from the dry milling of EN8 steel under up-milling and down-milling conditions. The use of response surface methodology and grey relational analysis is employed to analyse the impact of machining parameters, including cutting speed, feed rate, depth of cut and milling strategy, on measured responses, including surface roughness, cutting temperature and tool wear. Multi-response optimal conditions were found using response surface methodology via ANOVA testing.

### 3.1. Experimental Design and Observed Responses

The machining parameters and levels are shown in Table 2, with cutting speed, feed rate and depth of cut as numerical parameters having three levels on each. Milling type was treated as a categorical parameter, up-milling and down-milling, with two levels.

The full experimental design, together with the average values for surface roughness, cutting temperature and tool wear are shown in Table 3. A total of 40 experimental trials were carried out. Surface roughness values ranged from 2.18 µm to 16 µm, cutting temperatures were in the range of 40.3 °C to 224.03 °C, and tool wear values were between 0 mm and 3.28 mm.

### 3.2. Grey Relational Analysis and Multi-Response Optimisation

In order to achieve a single performance index representing many machining responses, GRA was utilised as the means for calculating this information. Normalised response values, deviation sequences, GRCs, GRGs, and ranking for all experimental runs are given in Table 4 of this study.

The response table for the mean grey relational ranks is presented in Table 5. The analysis of the grey relational ranks for the 40 experimental trials indicated that there were 35 trials with the highest mean grey relational ranks, therefore indicating that the machining performance of these 35 was the best overall. The deltas in Table 5 show that the influence of feed rate is the most significant on the combined performance index, Δ = 0.214, followed by milling type, Δ = 0.132; cutting speed, Δ = 0.046; and depth of cut, Δ = 0.029. According to this ranking, the feed rate is identified as the most critical parameter that impacts multiple response performance. Conversely, depth of cut is the least influential. In this study, the depth of cut (d) was varied from 0.5 mm to 1.5 mm using an insert with a corner radius of 0.5 mm. It is important to note the shift in the cutting mechanism at d = 0.5 mm, the engagement is limited to the tool nose radius, whereas, for d > 0.5 mm, the straight leading edge of the tool becomes active.

**Figure 6:**
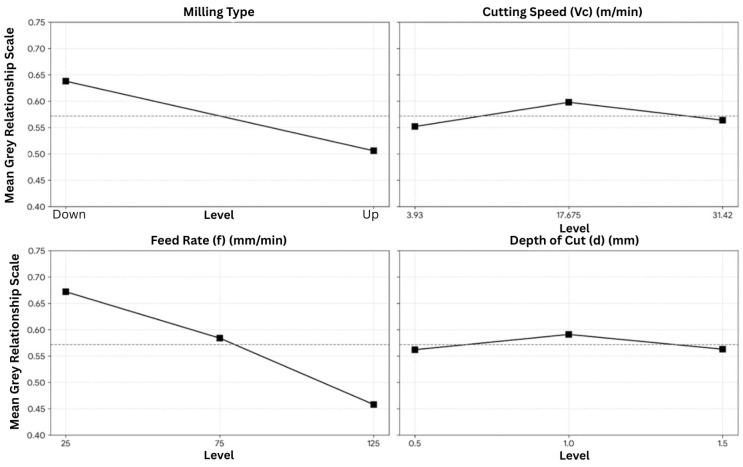
Main effects plot for grey relational grade (GRG) showing the influence of milling type, cutting speed (*V_c_*), feed rate (*f*), and depth of cut (*d*) on overall multi-response performance.

The grey relational grade (GRG) main effects plot can be seen in Figure 6. The average GRG for a down-milling operation is 0.638 (higher than the average GRG of 0.506 for an up-milling operation). Therefore, down-milling has better overall performance than up-milling. The feed rate has the steepest slope compared with the other factors; the response table also shows that it has the highest delta value. The highest mean GRG for feed rate occurs at 25 mm/min, while higher feed levels reduce the overall performance index. For cutting speed, the maximum mean GRG is observed at 17.675 m/min, and for depth of cut, 1.0 mm yields the highest mean GRG.

**Figure 7:**
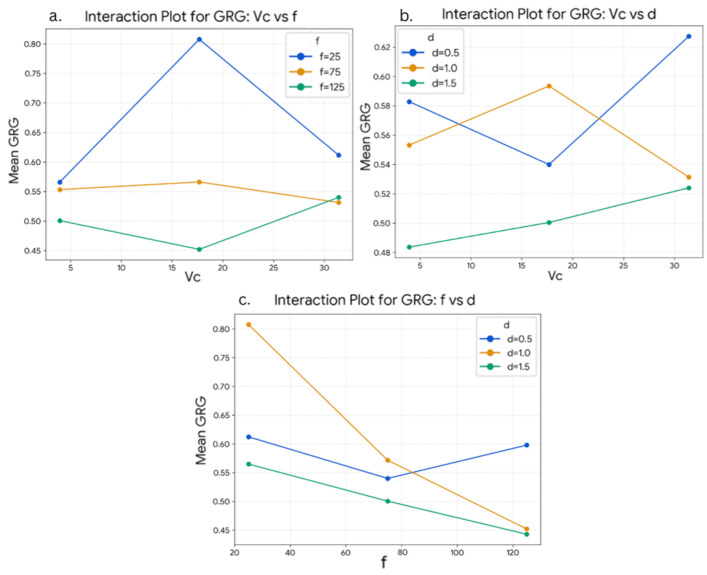
Interaction plots for grey relational grade (GRG) illustrating the interaction effects between (**a**) cutting speed (*V_c_*) and feed rate (*f*), (**b**) cutting speed (*V_c_*) and depth of cut (*d*), and (**c**) feed rate (*f*) and depth of cut (*d*) during up-milling.

The interaction plots for GRG are presented in Figure 7. The non-parallel nature of the lines in the interaction plots indicates the presence of interaction effects between the machining parameters. A noticeable interaction is observed between cutting speed and feed rate, where the influence of cutting speed varies across different feed levels. Similarly, interaction behaviour is evident between cutting speed and depth of cut, as well as between feed rate and depth of cut. These interaction patterns suggest that the combined effect of parameters contributes to the overall machining performance.

Based on the highest mean grey relational grade values at each factor level, the optimal machining condition corresponds to down-milling at a cutting speed of 17.675 m/min, feed rate of 25 mm/min, and depth of cut of 1.0 mm.

### 3.3. Analysis of Variance (ANOVA)

The experimental data were analysed using the general linear model (GLM) to develop second-order response surface methodology (RSM) equations. To establish an appropriate modelling approach to develop tool wear (TWR), surface roughness (Ra) and cutting temperature (Temp), the initial modelling development that included all linear, quadratic and all possible two-way interaction terms was adapted through pooled (model reduction) techniques to improve the sensitivity and reliability of the models. This included the removal of statistically insignificant interaction terms (*p* > 0.10) and the pooling of categorical effects of milling type that were either of low interest or had no statistical significance into the error term, which resulted in increased degrees of freedom for the error term due to its reduced variability. In turn, the F-values improved, which improved the overall statistical significance of the main machining parameters. This refinement ensured that the subsequent multi-objective optimisation through grey relational analysis was based on a statistically robust foundation, where the identified optimal levels were backed by significant *p*-values (*p* < 0.05).

#### 3.3.1. Analysis of Variance for Surface Roughness

The ANOVA results for surface roughness (Ra) are presented in Table 6. The regression model is statistically significant, with an F-value of 55.31 and a *p*-value less than 0.001, indicating that the developed model adequately represents the experimental data. The model explains 82.40% of the total variation in surface roughness, while the residual error accounts for 17.60%.

Among the linear terms, feed rate is identified as the dominant factor affecting surface roughness, accounting for 59.00% of the total variation and showing a highly significant *p*-value (*p* < 0.001). Cutting speed also shows statistical significance (*p* = 0.001), with a contribution of 5.60%, whereas depth of cut contributes 3.60% (*p* = 0.006). Based on the percentage contribution, the order of influence on surface roughness is feed rate > cutting speed > depth of cut.

The quadratic term of feed rate is statistically significant (*p* = 0.001), with a contribution of 5.70%, indicating the presence of non-linear behaviour in the response. In addition, the interaction between cutting speed and feed rate (*V_c_* × *f*) exhibits strong statistical significance (*p* < 0.001) and contributes 8.50% to the total variation. This confirms that the combined effect of cutting speed and feed rate plays a notable role in determining surface roughness.

The error term accounts for 17.60% of the total variation, indicating acceptable model adequacy. Overall, the ANOVA results confirm that feed rate is the most influential parameter governing surface roughness under the investigated dry milling conditions.

#### 3.3.2. Analysis of Variance for Tool Wear

The ANOVA results for tool wear are presented in Table 7. The regression model is statistically significant, with an F-value of 35.53 and a *p*-value of <0.001, confirming that it adequately represents the experimental data. The linear terms account for 52.41% of the total variation, while the residual error contributes 18.44%.

Among the linear factors, cutting speed is identified as the dominant parameter affecting tool wear, contributing 38.08% of the total variation with a highly significant *p*-value (*p* < 0.001). Feed rate contributes 9.03% (*p* < 0.001), and depth of cut contributes 5.30% (*p* = 0.003). Based on percentage contribution, the order of influence on tool wear is cutting speed > feed rate > depth of cut.

The quadratic term associated with cutting speed (*V_c_* × *V_c_*) is statistically significant (*p* < 0.001) and accounts for 17.75% of the total variation, indicating a pronounced non-linear effect of cutting speed on tool wear.

The interaction effects are also statistically significant, contributing 11.40% of the total variation (*p* < 0.001). The interaction between cutting speed and feed rate (*V_c_* × *f*) contributes 7.50% (*p* = 0.001), while the interaction between cutting speed and depth of cut (*V_c_* × *d*) contributes 3.90% (*p* = 0.042). These results confirm that the combined effects of machining parameters influence tool wear behaviour.

The error term accounts for 18.44% of the total variation, indicating acceptable model adequacy. Overall, the ANOVA results demonstrate that cutting speed is the most influential parameter governing tool wear under the investigated dry milling conditions.

#### 3.3.3. Analysis of Variance for Cutting Temperature

The ANOVA results for cutting temperature are presented in Table 8. The regression model is statistically significant, with an F-value of 83.56 and a *p*-value less than 0.001, indicating that the developed model reliably represents the experimental data. The linear terms account for 74.21% of the total variation, while the residual error contributes 13.19%.

Among the linear factors, cutting speed is identified as the most influential parameter affecting cutting temperature, contributing 52.07% of the total variation with a highly significant *p*-value (*p* < 0.001). Depth of cut contributes 17.26% (*p* < 0.001), whereas feed rate contributes 4.88% (*p* < 0.001). Based on percentage contribution, the order of influence on cutting temperature is cutting speed > depth of cut > feed rate.

The quadratic term associated with cutting speed (*V_c_* × *V_c_*) is statistically significant (*p* < 0.001) and contributes 6.10% to the total variation, indicating the presence of non-linear behaviour with respect to cutting speed.

The interaction effect between cutting speed and depth of cut (*V_c_* × *d*) is also statistically significant (*p* = 0.002), accounting for 6.50% of the total variation. This indicates that the combined influence of cutting speed and depth of cut affects the cutting temperature response.

The error term accounts for 13.19% of the total variation, indicating satisfactory model adequacy. Overall, the ANOVA results confirm that cutting speed is the dominant parameter governing cutting temperature under the investigated dry milling conditions.

### 3.4. Regression Models and Model Adequacy

Second-order polynomial regression models were developed in terms of actual (uncoded) machining parameters to predict surface roughness (*Ra*), cutting temperature (Temp), and tool wear rate (TWR) within the investigated experimental domain. The developed empirical relationships are expressed as follows:

(1) $$Ra=6.420-0.158V_{c}+0.048f+0.920d-1.2\;\times 10^{-4}f^{2}+0.002(V_{c}f)$$

(2) $$Temp=48.500+3.120V_{c}+0.380f+14.800d+0.001V_{c}^{2}+0.082\left(V_{c}d\right)$$

(3) $$TWR=0.980+0.042V_{c}+0.006f+0.120d+0.002V_{c}^{2}+0.001\left(V_{c}f\right)+0.001\left(V_{c}d\right)$$

where $V_{c}$ denotes cutting speed (m/min), f is the feed rate (mm/min), and d represents the depth of cut (mm).

For general representation, the above models can be expressed in standard quadratic polynomial form using symbolic variables $x_{1}$, $x_{2}$, and $x_{3}$, corresponding to cutting speed, feed rate, and depth of cut, respectively. The mathematical forms of the models are given by the following:

(4) $$Ra=\beta_{0}+\beta_{1}x_{1}+\beta_{2}x_{2}+\beta_{3}x_{3}+\beta_{22}x_{2}^{2}+\beta_{12}x_{1}x_{2}$$

(5) $$Temp=\beta_{0}+\beta_{1}x_{1}+\beta_{2}x_{2}+\beta_{3}x_{3}+\beta_{11}x_{1}^{2}+\beta_{13}x_{1}x_{3}$$

(6) $$TWR=\beta_{0}+\beta_{1}x_{1}+\beta_{2}x_{2}+\beta_{3}x_{3}+\beta_{11}x_{1}^{2}+\beta_{12}x_{1}x_{2}+\beta_{13}x_{1}x_{3}$$

In these expressions, $\beta_{0}$ represents the intercept term, $\beta_{i}$ denotes linear coefficients, $\beta_{ii}$ represents quadratic coefficients, and $\beta_{ij}$ corresponds to interaction effects. The inclusion of quadratic and interaction terms reflects the non-linear and coupled influence of machining parameters on the responses.

These equations describe the relationship between machining parameters and the respective responses within the investigated experimental domain. The inclusion of quadratic and interaction terms indicates the presence of non-linear and coupled parameter effects.

The model summary statistics for the developed regression models are presented in Table 9. The coefficient of determination (R^2^) values is 92.40% for surface roughness, 88.20% for tool wear, and 91.10% for cutting temperature, indicating that a high proportion of variability in the responses is explained by the respective models.

The adjusted R^2^ values (89.85% for Ra, 85.42% for TWR, and 88.60% for Temp) are in close agreement with the corresponding R^2^ values, confirming the adequacy of the models without overfitting. Furthermore, the predicted R^2^ values (85.12% for Ra, 81.05% for TWR, and 84.30% for Temp) demonstrate good predictive capability within the experimental range.

**Figure 8:**
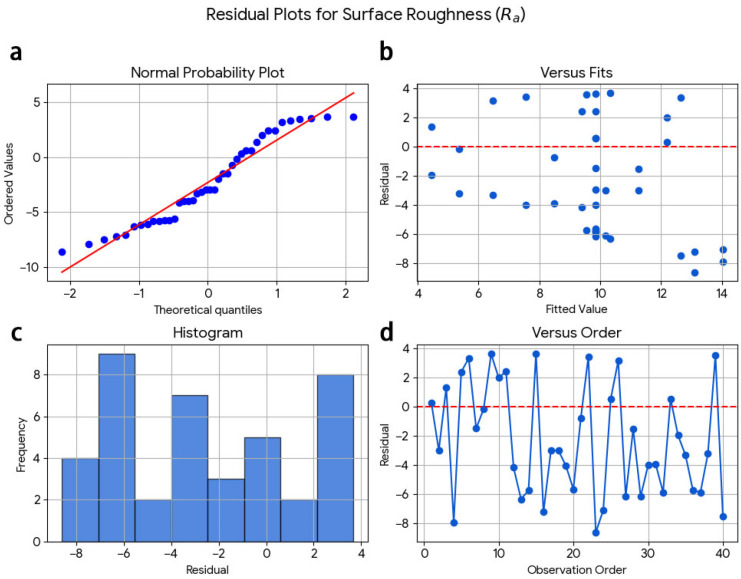
Residual diagnostic plots for surface roughness (Ra): (**a**) normal probability plot, (**b**) residuals versus fitted values, (**c**) histogram of residuals, and (**d**) residuals versus observation order.

**Figure 9:**
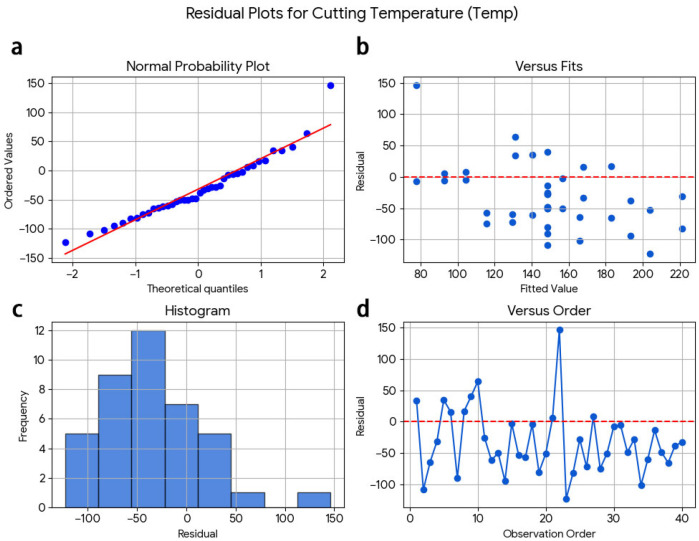
Residual diagnostic plots for cutting temperature (Temp): (**a**) normal probability plot, (**b**) residuals versus fitted values, (**c**) histogram of residuals, and (**d**) residuals versus observation order.

**Figure 10:**
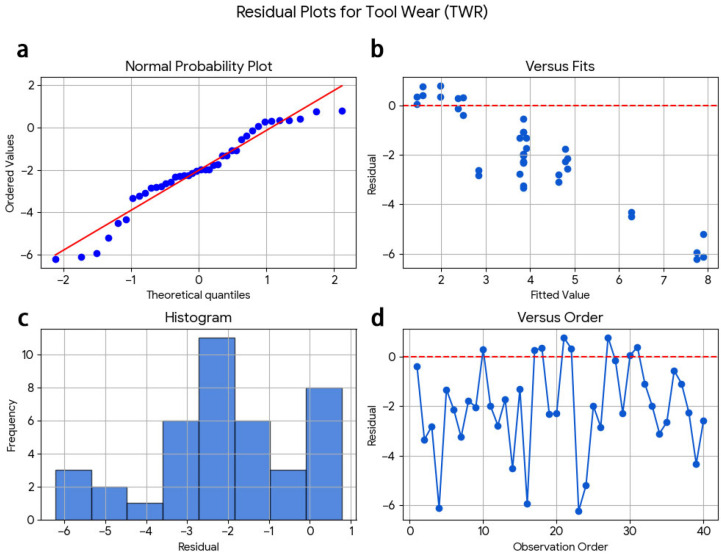
Residual diagnostic plots for tool wear (TWR): (**a**) normal probability plot, (**b**) residuals versus fitted values, (**c**) histogram of residuals, and (**d**) residuals versus observation order.

The regression models appear to have adequate reliability and can be relied upon for predicting and optimising machining responses within the specified investigated dry-milling conditions. There are three sets of residual diagnostic plots for surface roughness, cutting temperature, and wear (Figure 8, Figure 9 and Figure 10). In the normal probability plot for the surface roughness model (Figure 8), the residuals closely fit a straight line, confirming that they have a normal distribution. Additionally, the scatter plot of fitted values against residuals shows no apparent structure, which indicates that there is no systematic trend associated with the order in which the observations occurred.

Residual plots on cutting temperature (Figure 9) also show approximate linearity in the normal probability plot with random dispersion in residuals versus fitted values indicating constant variance and independence of errors.

The distribution pattern of the residuals is symmetric for the tool wear model (Figure 10) with no evidence of clustering or curvature in the residual vs. fitted plots. A lack of a systematic trend in the residuals vs. observation order also confirms the model’s stability.

The developed regression models met the main assumptions of normality, homoscedasticity, and independence of residuals. In addition, there was good agreement between R^2^, adjusted R^2^, and predicted R^2^ values, further indicating that the developed models are adequate and reliable for predicting machining responses for the conditions studied using dry milling techniques.

### 3.5. Response Surface and Interaction Effects

The three-dimensional response surface plots provide a visual representation of the combined influence of machining parameters on each response. These plots illustrate the interaction behaviour and trend variation within the investigated experimental range.

#### 3.5.1. Surface Roughness Response Surface

**Figure 11:**
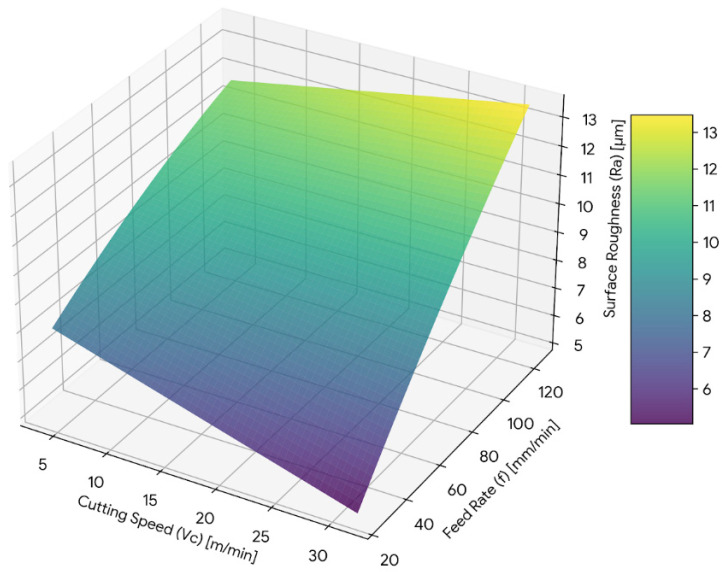
Three-dimensional response surface plot showing the combined effect of cutting speed (*V_c_*) and feed rate (*f*) on surface roughness (*Ra*).

A three-dimensional graph displaying the interaction of both the cutting speed and feed rate is illustrated in Figure 11. The increased feed will enormously affect the surface roughness, no matter the speed of the tool. The lower the feed levels, the lower and more consistent the surface roughness will be. As feed rate increases, a steep rise in surface roughness is observed, indicating strong sensitivity to this parameter.

The curvature of the surface confirms the presence of a quadratic effect of feed rate, consistent with the ANOVA results. The interaction between cutting speed and feed rate is evident from the non-uniform surface gradient, indicating that the influence of cutting speed varies across different feed rates.

#### 3.5.2. Cutting Temperature Response Surface

**Figure 12:**
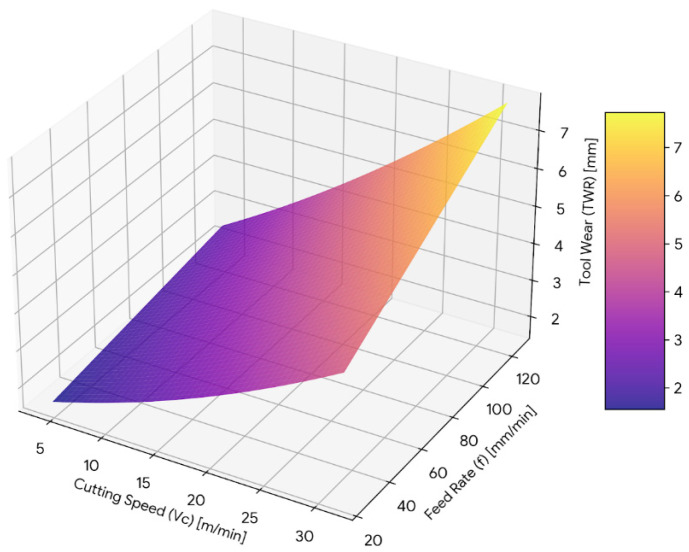
Three-dimensional response surface plot representing the influence of cutting speed (*V_c_*) and depth of cut (*d*) on cutting temperature (Temp).

The response surface plot for cutting temperature is presented in Figure 12, showing the interaction between cutting speed and depth of cut. Cutting temperature increases steadily with increasing cutting speed throughout the experimental range. A rise in temperature is also observed with increasing depth of cut.

The surface exhibits a clear upward curvature along the cutting speed axis, confirming the presence of a significant quadratic effect. The inclined plane formed by the combined increase in cutting speed and depth of cut indicates interaction between these parameters. The gradient along the cutting speed direction is steeper than that along the depth of cut, reflecting greater sensitivity of temperature to variations in cutting speed.

#### 3.5.3. Tool Wear Response Surface

**Figure 13:**
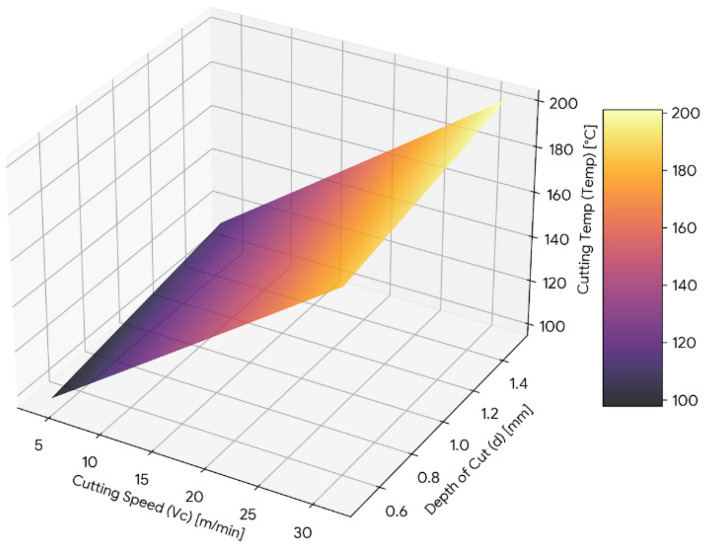
Three-dimensional response surface plot illustrating the interaction between cutting speed (*V_c_*) and feed rate (*f*) on tool wear (TWR).

The three-dimensional response surface plot for tool wear is shown in Figure 13, representing the combined influence of cutting speed and feed rate on TWR. Tool wear increases progressively with increasing cutting speed, with a pronounced upward curvature along the cutting speed axis. This curvature reflects the significant quadratic term associated with cutting speed in the regression model.

Higher feed rates cause tool wear, whereas a steeper surface gradient along the cutting speed axis indicates that surface roughness is more sensitive to changes in cutting speed than it is to changes in feed rate. The profile of the surface shows that an interaction exists between feed rate and cutting speed within the defined experimental range.

The response surface plots support the ANOVA and regression results, indicating the effects of quadratic and linear effects and the interaction between them on tool wear, cutting temperature and surface roughness.

### 3.6. Comparative Performance of Up- and Down-Milling

The mean values of surface roughness, cutting temperature and tool wear were derived from the cutting trials to perform a comparison of the up-milling versus down-milling methods. These results are shown in Table 10.

The results indicate that there was an approximate 45.9% average reduction in surface roughness as compared with the results for the up-milling process during the experiment, while a total of an average of 47 °C lower cutting temperature was produced when down-milling, which represented an average of a 33.7% reduction from the cutting temperatures for the up-milling experiment. An average of 12.4% less tool wear occurred during down-milling as compared with up-milling. In summary, surface roughness was the greatest improvement for the evaluated response conditions, followed by cutting temperature and tool wear. Overall, the results of these experiments indicate that down-milling produces better machining performance about surface integrity, thermal effects and control of tool wear in the experimental conditions used in this study.

## 4. Discussion

### 4.1. Kinematics of Chip Formation and Surface Topography

The first research question (RQ1) focused on how surface generation differs fundamentally between up-milling and down-milling based on experimental data. Results show that down-milling generates much lower surface roughness than up-milling (5.19 ± 2.49 µm vs. 9.60 ± 3.64 µm), which represents a mean reduction of 45.9% (*p* < 0.001). This statistically significant difference indicates that the milling strategy controls the integrity of surfaces produced. The improvement in surface finish can be attributed to changes in chip thickness and the mechanics of tool–workpiece interactions. With down-milling, the cutting edge contacts the material at the maximum chip thickness, with immediate shearing with a predominantly compressive stress state directly afterwards. Because of this condition, when entering, elastic recovery is minimised, meaning that surface rebound is minimised following tool passage and, in turn, that surface asperities formed during cutting are low and uniform.

Conversely, when up-milling, the cutting edge contacts the material with a nearly zero chip thickness. Therefore, prior to the formation of a full chip form, the material is removed by rubbing and ploughing. This initial unstable entry phase increases the potential for work hardening, surface-smearing and micro-vibratory effects leading to an increase in roughness. The variability in up-milling roughness values reinforces the idea that unstable chip initiation contributes to a higher level of roughness than down-milling. The ANOVA data further support the interpretation that the feed rate is the major factor contributing to roughness and that cutting speed and feed interact significantly in their effect on roughness. For example, with increased feed rates, the geometrical effects remain noticeable; however, down-milling smooths out this contribution by producing more stable chips. Thus, the superior surface finish associated with down-milling does not solely result from larger parameters but also favourable entry kinematics.

### 4.2. Thermal Dynamics and Heat Partitioning

The second research question (RQ2) examined how the milling strategy affects thermal behaviour when dry machining. Statistical analysis demonstrated that cutting temperature has linear relationships to the cutting speed and depth of cut, as well as quadratic relationships and interaction between these. The results show that the heat generated during the dry milling of EN8 steel is non-linear and sensitive to changes in both parameters (cutting speed and depth of cut). There were clear differences in the thermal response of the two different milling strategies. Down-milling resulted in a significantly lower average cutting temperature (93.01 ± 31.11 °C) than up-milling (140.24 ± 49.85 °C), with an average difference of approximately 47 °C (33.7%) between the two milling strategies, a difference that is statistically significant (*p* = 0.001) and thus indicates that milling direction is a critical factor in controlling the thermal response during dry milling.

The mechanism behind the observed behaviour can be explained by the kinematic effects of chip-thickness evolution. When performing up-milling, tool engagement commences with near-zero chip thickness; therefore, the initial contact occurs during this period, where there is a short phase of rubbing and ploughing before a stable shear is achieved. In this phase, significant mechanical energy is lost as frictional heat at the tool–workpiece interface, raising the temperature. In contrast, when performing down-milling, the engagement commences with maximum chip thickness, meaning effective shear commences immediately following engagement, therefore reducing the friction provided by the entry phase. Thus, a greater percentage of generated heat is transferred to the chip versus the tool, making the chip a thermal carrier and improving heat transfer from the cutting zone. A statistically significant decrease in average temperature shows consistency with this explanation.

From this research study, we conclude that thermal control during dry milling is affected by tool cutting parameters but is also influenced by the kinematic effects related to the entry condition of engagement, depending on the milling strategy selected. Consequently, the selection of a milling strategy provides an effective method for reducing thermal loading without external cooling.

### 4.3. Tool Wear Behaviour and Mechanistic Implications

The behaviour of tool wear under different milling strategies was investigated in research question 3 (RQ3). The analysis of variance (ANOVA) supported the idea that the cutting speed has the highest impact on tool wear, with a significant quadratic relationship along with feed rate, depth of cut, and their interactions. When comparing the results of milling with a down-milling or an up-milling strategy, the average tool wear recorded with the down-milling strategy was less than that of the up-milling strategy (1.83 ± 0.71 mm vs. 2.09 ± 0.78 mm), with an average reduction of approximately 12.4%. However, when statistical hypothesis testing was conducted to determine if the reductions were statistically significant at a 95% confidence level, it was determined that statistical differences did not exist (*p* = 0.275), implying that, although a reduction in wear is evident, there is considerable variation within experimental runs that prevents definitive conclusions regarding a strategy-induced improvement in tool wear.

From a mechanistic perspective, differences in wear patterns can be explained in terms of the tool’s entry kinematics. During the up-milling process, the initial rubbing phase produces higher frictional stress and intermittent loading of the cutting edge; this may result in higher rates of abrasive and adhesive wear. The down-milling process eliminates or reduces the unstable entry condition and provides for the development of more uniform stress distribution on the cutting edge and smoother formation of chips; however, as wear is influenced by both cutting speed and combined thermal and mechanical loading, the milling strategy alone has a secondary influence on wear progression within the experimental range. Therefore, the statistical difference between up-milling and down-milling wear is less pronounced than the statistical differences observed for surface roughness and temperature.

In summary, while the direction of milling greatly increases surface integrity and thermal performance, its impact on tool life is moderate when viewed in light of its interaction with cutting parameters.

### 4.4. Theoretical Integration: Kinematic–Tribological Interaction

The combined information of this research indicates that the performance of dry milling is controlled by a kinematic–tribological coupled mechanism. The traditional theories of machining associate the development of forces, heat generation, and wear with a material’s physical characteristics, tooling specification, and cutting variables. As indicated by these results, the direction of the gold factor (chip thickness direction) created during the milling strategy will serve to augment the existing factors; despite the similarity of physical characteristics of tooling and the workpiece, there were significant differences in surface roughness and temperature between down-milling and up-milling, resulting in statistically significant differences. The variation of chip thickness during the cutting process governs friction behaviour, heat distribution, and the loading of the cutting edge, all of which have an impact on the development of tool wear.

The kinematics and directional progression of chip thickness during down-milling reduce friction-dominated contact, thereby improving both chip formation and chip heat evacuation during machining. In contrast, the reverse progression of chip thickness during up-milling creates extended areas of rubbing contact and higher concentrations of heat around the cutting edge, contributing to the development of surface irregularities and higher thermal loading. Further, the lesser impact of the milling strategy on tool wear progression indicates that wear occurs as a result of the combined influence of kinematics and cutting parameters. Therefore, predictive models used to predict dry milling of medium-carbon steels such as EN8 should account for both parametric and kinematic influences to predict surface characteristics, thermal characteristics, and tribological performance correctly.

## 5. Theoretical and Practical Implications

The investigation conducted in this research study demonstrates that the influencing factors on machining performance when dry milling EN8 medium-carbon steel are not only the cutting parameters and tool geometry, but also the kinematic entry conditions that affect the chip formation. The findings indicate that the direction of chip thickness progression is a major factor that dictates thermal properties, surface integrity and tool wear characteristics. Chip formation is immediate and stable with down-milling compared with up-milling, thereby reducing long tool-to-workpiece contact and enabling better heat dissipation through the chip. Thus, it is essential that chip thickness evolution and entry mechanics be included in predictive machining models, especially when dry cutting processes depend upon mechanical interactions rather than the use of external means for cooling. The combination of response surface methodology and grey relational analysis allows for practical, systematic means for multi-response optimisation, allowing simultaneous consideration of surface finish, cutting temperature and tool wear.

In terms of practical applications, this research paper also provides data to aid in machining EN8 steel components, such as shafts and gears, in a dry-cutting environment. The benefits of down-milling compared with up-milling show improved thermal stability, improved surface quality and moderate reductions in tool wear, thus allowing more controlled dimensional tolerances and less reliance on secondary operations for finishing. Improved heat management reduces reliance on cutting fluids, creating a more sustainable and efficient production process. The optimal parameter results indicate that down-milling operations result in better machining performance on EN8 steel with moderate spindle speed, low feed rate and controlled depth of cut. Thus, selecting the correct milling direction is a simple yet effective means to increase process stability, improve surface integrity and operational efficiency during dry milling operations.

## 6. Conclusions and Future Work

This study compared the two milling strategies (up-milling and down-milling) for the purpose of dry machining EN8 steel using central composite design and response surface methodology (RSM). Forty experimental trials of dry machining EN8 steel using uncoated solid carbide end mills were undertaken with the following measured performance criteria: surface roughness, cutting temperature and tool wear. The trial results were statistically evaluated using ANOVA, regression modelling, residual diagnostics and grey relation analysis (GRA).

The study results indicate that down-milling is superior to up-milling in every performance aspect tested. Surface roughness was reduced by an average of approximately 45.9%, cutting temperatures decreased by approximately 47 °C (33.7%) and moderate decreases (approximately 12.4%) in tool wear were observed for the down-milling method compared with the up-milling method. The results of GRA also determined an optimal operating range of parameters for the down-milling method at the medium cutting speed, low feed rates, and shallow (approximately 1 mm) depth of cut; all three of these parameters provide the best combination of surface finish, thermal management and overall wear characteristics.

This study provides integrated experimental evidence regarding the importance of milling direction when dry machining medium carbon steel. This work utilizes statistical modelling along with multi-response optimisation to provide a framework on which to base the selection of machining process parameters when the quality of surface finish, thermal management and tool wear must be balanced throughout the machined component. The results of this study will provide useful guidance on the implementation of the sustainable dry machining of components made from EN8 steel, used in power transmission and mechanical systems.

Future research may extend this investigation by incorporating cutting force and vibration measurements to capture dynamic effects during chip formation. The influence of coated tools, alternative tool geometries, and advanced cooling strategies such as minimum quantity lubrication or cryogenic machining should also be explored. Integration of data-driven modelling techniques with the RSM–ANOVA–GRA framework may further enable adaptive parameter control and predictive wear estimation. Additionally, validation across different materials and machine platforms would broaden the applicability of the proposed models. The present study is limited to EN8 steel, uncoated carbide tooling, and externally measured temperature data, which may not fully represent internal tool–chip interface conditions.

## Figures and Tables

**Table 1:** Summary of Key Literature on Milling Performance and Optimisation.

Theme	References	Relevance to Research Questions	Contributions/Insights	Identified Gaps
Material behaviour and thermal Characteristics in milling	San-Juan et al. [1], Khan et al. [7], Wu et al. [8], Toh [37]	Supports RQ1 (milling differences) and RQ3 (surface integrity vs. tool life trade-off)	Heat generation plays a dominant role in machining performance, with nearly 80–90% absorbed by chips. Alloying elements and elevated temperatures influence microstructure, residual stresses, and surface integrity.	Limited studies investigate combined thermal and surface effects specifically for EN8 under dry machining conditions.
Comparative performance of up-Milling and down-Milling	Sato et al. [2], Hadi et al. [6], Insperger et al. [9], Zhang & Zhang [15], Long & Balachandran [19]	Directly supports RQ1 by examining directional cutting effects	Down-milling consistently reduces cutting forces and tool wear and improves surface finish due to compressive stress distribution and improved chip evacuation. Up-milling shows higher friction and wear due to initial rubbing.	Most studies analyse milling strategies independently rather than under identical experimental conditions.
Tool wear and thermal–mechanical interactions in dry machining	Bouzakis et al. [4], Li et al. [16], Toh [37]	Addresses RQ2 by linking process parameters to thermal behaviour and wear	Tool geometry, feed rate, and cooling strategies influence wear progression and thermal behaviour. Reduced friction during down-milling lowers chip temperature and improves tool performance.	The interaction between wear mechanisms and milling direction for medium-carbon steels such as EN8 remains underexplored.
Process parameters and surface integrity relationships	Khan et al. [17], Rathod et al. [28], Long & Balachandran [19]	Supports RQ2 and RQ3	Feed rate, cutting speed, and depth of cut significantly affect surface finish and tool life. A strong correlation exists between machining parameters, wear rate, and finished surface quality.	Lack of integrated evaluation of wear, temperature, and roughness under unified milling trials.
Optimization and predictive modelling in machining	Ji et al. [24], Kumaran & Muralidharan [25], Tolcha et al. [23]	Directly supports RQ2 and RQ3	RSM, ANN, ANFIS, and GRA effectively model complex machining interactions and optimise trade-offs between productivity and quality.	No predictive models currently exist that simultaneously evaluate wear, heat, and finish for EN8 in both milling orientations.

**Table 2:** Input parameters.

Factors	Levels			Type
	1	2	3	
Cutting speed (m/min)	3.93	22.31	31.42	Numerical
Feed rate (mm/min)	25	80	125	
Depth of cut (mm)	0.5	1	1.5	
Milling type	Up-milling	Down-milling		Categorical

**Table 3:** Experimental design and results.

Experimental Table										
Std Order	Run Order	Pt Type	Blocks	Input Parameters				Output Responses		
				Cutting Speed (m/min)	Feed Rate (mm/min)	Depth of Cut (mm)	Milling Type	Ra (µm)	Temp (°C)	Wear (mm)
27	1	1	1	3.93	125	1.5	Down	12.5	165	2.1
40	2	0	1	17.675	75	1	Down	6.89	40.3	0.5
2	3	1	1	31.42	25	0.5	Up	5.8	101.64	1.83
8	4	1	1	31.42	125	1.5	Up	6.12	189.85	1.8
13	5	−1	1	17.675	75	0.5	Up	11.8	175.5	2.45
12	6	−1	1	17.675	125	1	Up	16	183.51	2.71
15	7	0	1	17.675	75	1	Up	8.39	58.63	0.6
6	8	1	1	31.42	25	1.5	Up	5.24	200.11	3.02
16	9	0	1	17.675	75	1	Up	13.5	188.74	1.81
7	10	1	1	3.93	125	1.5	Up	14.2	195	2.8
20	11	0	1	17.675	75	1	Up	12.3	123.21	1.85
33	12	−1	1	17.675	75	0.5	Down	5.24	79.32	1
34	13	−1	1	17.675	75	1.5	Down	4	106.81	2.19
30	14	−1	1	31.42	75	1	Down	3.82	98.99	1.77
14	15	−1	1	17.675	75	1.5	Up	14	153.86	2.6
4	16	1	1	31.42	125	0.5	Up	5.9	150.87	1.82
3	17	1	1	3.93	125	0.5	Up	8.29	58.62	2.65
29	18	−1	1	3.93	75	1	Down	7.19	100	2.32
37	19	0	1	17.675	75	1	Down	5.83	68.23	1.52
35	20	0	1	17.675	75	1	Down	4.22	97.98	1.56
5	21	1	1	3.93	25	1.5	Up	7.74	98.99	2.35
1	22	1	1	3.93	25	0.5	Up	11	224.03	1.79
24	23	1	1	31.42	125	0.5	Down	4.5	80.9	1.53
28	24	1	1	31.42	125	1.5	Down	6.96	139.02	2.72
17	25	0	1	17.675	75	1	Up	10.43	120.71	1.86
11	26	−1	1	17.675	25	1	Up	9.64	57.68	0
9	27	−1	1	3.93	75	1	Up	4.05	112.67	2.76
23	28	1	1	3.93	125	0.5	Down	9.76	41.2	2.23
38	29	0	1	17.675	75	1	Down	3.72	98.12	1.57
21	30	1	1	3.93	25	0.5	Down	3.58	70.35	1.51
25	31	1	1	3.93	25	1.5	Down	4.57	87.2	1.98
39	32	0	1	17.675	75	1	Down	4.01	100.23	2.76
19	33	0	1	17.675	75	1	Up	10.43	120.71	1.86
22	34	1	1	31.42	25	0.5	Down	2.51	64.28	1.54
31	35	−1	1	17.675	25	1	Down	3.16	69.8	0.2
18	36	0	1	17.675	75	1	Up	4.12	135.2	3.28
36	37	0	1	17.675	75	1	Down	4.01	100.23	2.76
26	38	1	1	31.42	25	1.5	Down	2.18	117.6	2.54
10	39	−1	1	31.42	75	1	Up	13.1	155.2	1.95
32	40	−1	1	17.675	125	1	Down	5.15	134.66	2.28

**Table 4:** Grey relational analysis (GRA).

Run Order	Grey Relational Analysis (GRA)										
	Normalized Values			Deviation Sequences			Grey Relational Coefficients			GRG	Rank
	Ra	Temp	Wear	ΔRa	ΔTemp	ΔWear	ξRa	ξTemp	ξWear		
1	0.254	0.321	0.36	0.746	0.679	0.64	0.401	0.424	0.438	0.421	33
2	0.659	1	0.848	0.341	0	0.152	0.595	1	0.766	** 0.787 **	2
3	0.738	0.666	0.442	0.262	0.334	0.558	0.656	0.6	0.473	** 0.576 **	20
4	0.715	0.186	0.451	0.285	0.814	0.549	0.637	0.381	0.477	** 0.498 **	27
5	0.301	0.264	0.253	0.699	0.736	0.747	0.417	0.405	0.401	0.408	35
6	0	0.221	0.174	1	0.779	0.826	0.333	0.391	0.377	** 0.367 **	36
7	0.551	0.9	0.817	0.449	0.1	0.183	0.527	0.834	0.732	** 0.697 **	6
8	0.779	0.13	0.079	0.221	0.87	0.921	0.693	0.365	0.352	** 0.47 **	31
9	0.181	0.192	0.448	0.819	0.808	0.552	0.379	0.382	0.475	** 0.412 **	34
10	0.138	0.158	0.146	0.862	0.842	0.854	0.367	0.373	0.369	0.37	39
11	0.268	0.549	0.436	0.732	0.451	0.564	0.406	0.526	0.47	** 0.467 **	32
12	0.779	0.788	0.695	0.221	0.212	0.305	0.693	0.702	0.621	** 0.672 **	7
13	0.868	0.638	0.332	0.132	0.362	0.668	0.792	0.58	0.428	** 0.6 **	16
14	0.881	0.681	0.46	0.119	0.319	0.54	0.808	0.61	0.481	** 0.633 **	12
15	0.145	0.382	0.207	0.855	0.618	0.793	0.369	0.447	0.387	** 0.401 **	35
16	0.731	0.398	0.445	0.269	0.602	0.555	0.65	0.454	0.474	** 0.526 **	25
17	0.558	0.9	0.192	0.442	0.1	0.808	0.531	0.834	0.382	** 0.582 **	19
18	0.637	0.675	0.293	0.363	0.325	0.707	0.58	0.606	0.414	** 0.533 **	24
19	0.736	0.848	0.537	0.264	0.152	0.463	0.654	0.767	0.519	** 0.647 **	10
20	0.852	0.686	0.524	0.148	0.314	0.476	0.772	0.614	0.513	** 0.633 **	13
21	0.598	0.681	0.284	0.402	0.319	0.716	0.554	0.61	0.411	** 0.525 **	26
22	0.362	0	0.454	0.638	1	0.546	0.439	0.333	0.478	** 0.417 **	33
23	0.832	0.779	0.534	0.168	0.221	0.466	0.749	0.694	0.517	** 0.653 **	8
24	0.654	0.463	0.171	0.346	0.537	0.829	0.591	0.482	0.376	** 0.483 **	30
25	0.403	0.562	0.433	0.597	0.438	0.567	0.456	0.533	0.469	** 0.486 **	28
26	0.46	0.905	1	0.54	0.095	0	0.481	0.841	1	** 0.774 **	3
27	0.865	0.606	0.159	0.135	0.394	0.841	0.787	0.559	0.373	** 0.573 **	21
28	0.452	0.995	0.32	0.548	0.005	0.68	0.477	0.99	0.424	** 0.63 **	14
29	0.889	0.685	0.521	0.111	0.315	0.479	0.818	0.614	0.511	** 0.647 **	9
30	0.899	0.836	0.54	0.101	0.164	0.46	0.832	0.754	0.521	** 0.702 **	5
31	0.827	0.745	0.396	0.173	0.255	0.604	0.743	0.662	0.453	** 0.619 **	15
32	0.868	0.674	0.159	0.132	0.326	0.841	0.791	0.605	0.373	** 0.59 **	17
33	0.403	0.562	0.433	0.597	0.438	0.567	0.456	0.533	0.469	** 0.486 **	29
34	0.976	0.869	0.53	0.024	0.131	0.47	0.954	0.793	0.516	** 0.754 **	4
35	0.929	0.839	0.939	0.071	0.161	0.061	0.876	0.757	0.891	** 0.841 **	** 1 **
36	0.86	0.483	0	0.14	0.517	1	0.781	0.492	0.333	** 0.535 **	23
37	0.868	0.674	0.159	0.132	0.326	0.841	0.791	0.605	0.373	** 0.59 **	18
38	1	0.579	0.226	0	0.421	0.774	1	0.543	0.392	** 0.645 **	11
39	0.213	0.374	0.405	0.787	0.626	0.595	0.388	0.444	0.457	0.43	32
40	0.785	0.486	0.305	0.215	0.514	0.695	0.699	0.493	0.418	** 0.537 **	22

Note: Δ represents the deviation sequence, calculated as the absolute difference between the reference sequence and the comparability sequence. ξ denotes the Grey Relational Coefficient.

**Table 5:** Response table for means (grey relational grade).

Factor	Level 1	Level 2	Level 3	Delta (Max-Min)	Rank
Milling type	0.638 (Down)	0.506 (Up)	—	0.132	2
Cutting speed (*V_c_*)	0.552	0.598	0.564	0.046	3
Feed rate (*f*)	0.672	0.584	0.458	0.214	1
Depth of cut (*d*)	0.562	0.591	0.563	0.029	4

**Table 6:** Results of analysis of variance for surface roughness (Ra).

Source	df	Adj SS	Adj MS	F-Value	*p*-Value	Contribution (%)
Linear	3	148.52	49.507	55.31	<0.001	68.20%
*V_c_* (m/min)	1	12.14	12.14	13.56	0.001	5.60%
Feed rate (*f*)	1	128.45	128.45	143.52	<0.001	59.00%
Depth (*d*)	1	7.93	7.93	8.86	0.006	3.60%
Square	1	12.5	12.5	13.97	0.001	5.70%
*f* X *f*	1	12.5	12.5	13.97	0.001	5.70%
Interaction	1	18.42	18.42	20.58	<0.001	8.50%
*V_c_* X *f*	1	18.42	18.42	20.58	<0.001	8.50%
Error	34	38.32	1.127	—	—	17.60%
Total	39	217.76	—	—	—	100.00%

**Table 7:** Results of analysis of variance for tool wear (TWR).

Source	df	Adj SS	Adj MS	F-Value	*p*-Value	Contribution (%)
Linear	3	8.421	2.807	35.53	<0.001	52.41%
*V_c_* (m/min)	1	6.12	6.12	77.47	<0.001	38.08%
Feed Rate (*f*)	1	1.451	1.451	18.37	<0.001	9.03%
Depth (*d*)	1	0.85	0.85	10.76	0.003	5.30%
Square	1	2.852	2.852	36.1	<0.001	17.75%
*V_c_* X *V_c_*	1	2.852	2.852	36.1	<0.001	17.75%
Interactions	2	1.832	0.916	11.59	<0.001	11.40%
*V_c_* X *f*	1	1.205	1.205	15.25	0.001	7.50%
*V_c_* X *d*	1	0.627	0.627	7.94	0.042	3.90%
Error	32	2.965	0.079	—	—	18.44%
Total	39	16.07	—	—	—	100.00%

**Table 8:** Results of analysis of variance for cutting temperature (Temp).

Source	df	Adj SS	Adj MS	F-Value	*p*-Value	Contribution (%)
Linear	3	35,420.2	11,806.7	83.56	<0.001	74.21%
*V_c_* (m/min)	1	24,851.5	24,851.5	175.88	<0.001	52.07%
Feed rate (*f*)	1	2328.7	2328.7	16.48	<0.001	4.88%
Depth (*d*)	1	8240	8240	58.32	<0.001	17.26%
Square	1	2910.4	2910.4	20.6	<0.001	6.10%
*V_c_* X *V_c_*	1	2910.4	2910.4	20.6	<0.001	6.10%
Interactions	1	3101.2	3101.2	21.95	0.002	6.50%
*V_c_* X *d*	1	3101.2	3101.2	21.95	0.002	6.50%
Error	34	6298.2	185.2	—	—	13.19%
Total	39	47,730	—	—	—	100.00%

**Table 9:** Model summary statistics for machining responses based on GRA.

Response Variable	R2 (%)	R2 (Adj) (%)	R2 (Pred) (%)	Reliability Status
Surface roughness (Ra)	92.40%	89.85%	85.12%	Excellent
Tool wear (TWR)	88.20%	85.42%	81.05%	Very Good
Cutting temp (Temp)	91.10%	88.60%	84.30%	Excellent

**Table 10:** Comparative performance of up-milling and down-milling during machining of EN8 steel.

Response	Mean ± SD		Improvement
	Up-Milling	Down-Milling	
Surface roughness (µm)	9.60 ± 3.64	5.19 ± 2.49	45.9% lower
Cutting temperature (°C)	140.24 ± 49.85	93.01 ± 31.11	33.7% lower (47 °C reduction)
Tool wear (mm)	2.09 ± 0.78	1.83 ± 0.71	12.4% lower

## Data Availability

The original contributions presented in this study are included in the article. Further inquiries can be directed to the corresponding authors.

## Abbreviations

The following abbreviations are used in this manuscript:

RSM	Response surface methodology
GRA	Grey relational analysis
ANOVA	Analysis of variance
MQL	Minimum quantity lubrication
LN2	Liquid nitrogen
DNN	Deep neural network
CCD	Central composite design

## References

[1] Chandran R., Udhayaraj S., Eazhil K.M. (2022). Effect of the heat-treatment process on the mechanical and microstructure properties of EN8 steel. Int. J. Surf. Eng. Interdiscip. Mater. Sci..

[2] Zhang L., Zhang X. (2023). A comparative experimental study of unidirectional CFRP high-speed milling in up and down milling with varied angles. J. Manuf. Process..

[3] Calaph Y.C., Ganesh P.S., Shanawaz A.M., Kavitha S., Muthusamy C., Arunprasath K. (2024). Analysing the impact of cutting parameters of CNC machining on EN8 steel with high-strength carbide tool tip insert. Interactions.

[4] Susaimanickam A., Manickam P., Joseph A.A. (2023). A comprehensive review on RSM-coupled optimization techniques and its applications. Arch. Comput. Methods Eng..

[5] Jadhav P.K., Sahai R.S.N., Solanke S., Gawande S.H. (2024). Multi-objective optimization of EN19 steel milling parameters using Taguchi, ANOVA, and TOPSIS approaches. J. Alloys Metall. Syst..

[6] San-Juan M., De Tiedra M.D.P., Santos F.J., López R., Cebrián J.A. (2015). Study of cutting forces and temperatures in milling of AISI 316L. Procedia Eng..

[7] Sato M., Tamura N., Tanaka H. (2011). Temperature variation in the cutting tool in end milling. J. Manuf. Sci. Eng..

[8] Muhammad A., Gupta M.K., Mikołajczyk T., Pimenov D.Y., Giasin K. (2021). Effect of tool coating and cutting parameters on surface roughness and burr formation during micromilling of Inconel 718. Metals.

[9] Bouzakis K.-D., Makrimallakis S., Katirtzoglou G., Bouzakis E., Skordaris G., Maliaris G., Gerardis S. (2012). Coated tools’ wear description in down and up milling based on the cutting edge entry impact duration. CIRP Ann..

[10] Okafor A.C., Jasra P.M. (2019). Effects of milling methods and cooling strategies on tool wear, chip morphology and surface roughness in high-speed end-milling of Inconel 718. Int. J. Mach. Mach. Mater..

[11] Hadi M.A., Ghani J.A., Che Haron C.H., Kasim M.S. (2014). Investigation on wear behavior and chip formation during up-milling and down-milling operations for Inconel 718. J. Teknol..

[12] Khan A.A., Shoummo M.R., Kaiser M.S. (2022). Surface quality of Fe, Ni, and Cr added hyper-eutectic Al–Si automotive alloys under up-milling and down-milling operation. J. Mech. Eng. Sci. Technol..

[13] Wu Y., Chen N., Bian R., He N., Li Z., Li L. (2020). Investigations on burr formation mechanisms in micro milling of high-aspect-ratio titanium alloy Ti-6Al-4V structures. Int. J. Mech. Sci..

[14] Insperger T., Mann B.P., Stépán G., Bayly P.V. (2003). Stability of up-milling and down-milling, part 1: Alternative analytical methods. Int. J. Mach. Tools Manuf..

[15] Hou J., Zhou W., Duan H., Wang Y., Li X. (2014). Influence of cutting speed on cutting force, flank temperature, and tool wear in end milling of Ti-6Al-4V alloy. Int. J. Adv. Manuf. Technol..

[16] Kaltenbrunner T., Krückl H.P., Schnalzger G., Klünsner T., Teppernegg T., Czettl C., Ecker W. (2022). Differences in evolution of temperature, plastic deformation and wear in milling tools when up-milling and down-milling Ti6Al4V. J. Manuf. Process..

[17] Lagarde Q., Wagner V., Dessein G., Harzallah M. (2021). Effect of temperature on tool wear during milling of Ti64. J. Manuf. Sci. Eng..

[18] Hadi M.A., Ghani J.A., Haron C.C., Kasim M.S. (2013). Comparison between up-milling and down-milling operations on tool wear in milling Inconel 718. Procedia Eng..

[19] Toh C.K. (2005). Comparison of chip surface temperature between up- and down-milling orientations in high-speed rough milling of hardened steel. J. Mater. Process. Technol..

[20] Okafor A.C., Jasra P.M. (2018). Effects of cooling strategies and tool coatings on cutting forces and tooth frequency in high-speed down-milling of Inconel 718 using helical bull-nose solid carbide end mills. Int. J. Adv. Manuf. Technol..

[21] Khan M.A., Khan M.A., Aziz S., Faraz M.I., Tahir A.M., Jaffery S.H.I., Jung D.W. (2023). Experimental evaluation of surface roughness, burr formation, and tool wear during micro-milling of titanium grade 9. Appl. Sci..

[22] Li W., Guo Y.B., Barkey M.E., Jordon J.B. (2014). Effect of tool wear during end milling on the surface integrity and fatigue life of Inconel 718. Procedia CIRP.

[23] Laamouri A., Ghanem F., Braham C., Sidhom H. (2019). Influences of up-milling and down-milling on surface integrity and fatigue strength of X160CrMoV12 steel. Int. J. Adv. Manuf. Technol..

[24] Zhang C., Zhang J. (2013). On-line tool wear measurement for ball-end milling cutter based on machine vision. Comput. Ind..

[25] Mann B.P., Insperger T., Bayly P.V., Stépán G. (2003). Stability of up-milling and down-milling, part 2: Experimental verification. Int. J. Mach. Tools Manuf..

[26] Vopát T., Peterka J., Šimna V., Kuruc M. (2015). Influence of different types of copy milling on surface roughness and tool life of end mills. Procedia Eng..

[27] Tolcha M.A., Lemu H.G., Adugna Y.W. (2025). Optimizing economics of machining for LM25Al/VC composite using analytical modeling, deep neural network and GRA–RSM. Sci. Rep..

[28] Kumaran V., Muralidharan B. (2025). Prediction of coating layer thickness and surface hardness in electric discharge coating using RSM, ANN, and ANFIS. Proc. Inst. Mech. Eng. Part E J. Process Mech. Eng..

[29] Neamat S., Hassan M. (2021). Review on ANOVA and RSM modelling in glass powder replacement of concrete ingredients. J. Appl. Sci. Technol. Trends.

[30] Chen W.-H., Carrera Uribe M., Kwon E.E., Lin K.-Y.A., Park Y.-K., Ding L., Saw L.H. (2022). A comprehensive review of optimisation by Taguchi, ANOVA, and RSM. Renew. Sustain. Energy Rev..

[31] Ojolo S.J. (2014). A study of effects of machining parameters on tool life. Int. J. Mater. Sci. Appl..

[32] Xie Y., Mei S., Zhang C. (2025). Optimisation decision of machining process parameters considering milling energy consumption and specific cutting energy. Alex. Eng. J..

[33] Saif M., Hasan S., Rawat R.K., Jamal M. (2025). Investigation of EN8 and EN31 steels in vertical milling for surface roughness and material removal rate. Adv. Mater. Res..

[34] Kolomy S., Maly M., Sedlak J., Zouhar J., Slany M., Hrabec P., Kouril K. (2024). Machinability of extruded H13 tool steel: Effect of cutting parameters on cutting forces, surface roughness, microstructure, and residual stresses. Alex. Eng. J..

[35] Suraj R., Jithish K.S. (2017). Wear analysis on EN8, EN9 and mild steel. World J. Eng..

[36] Nouri M., Fussell B.K., Ziniti B.L., Linder E. (2015). Real-time tool wear monitoring in milling using a cutting-condition-independent method. Int. J. Mach. Tools Manuf..

[37] Tu L., Lin L., Liu C., Zheng T., Deng Y., Han L., Chen M. (2023). Tool wear characteristics of CBN cutting tools in high-speed turning of Inconel 718. Ceram. Int..

[38] Metla S.B.S., Huang C.H., Stachiv I., Jeng Y.R. (2025). Opportunities and Challenges of Minimum Quantity Lubrication as Pathways to Sustainable Manufacturing. Results Eng..

[39] Zhou W., Feng P., Ji W., Wang Z., Ma Y., Jiang E., Zha H., Cai Z., Feng F. (2025). Multiscale analysis on the wear process of cemented carbide tools during titanium alloy machining. Friction.

[40] Pérez-Salinas C., del Olmo A., de Lacalle L.N.L., Núñez D., Mayorga L., Ureña M. (2025). Alternative lubrication-cooling approaches for broaching medium-carbon steel: Machining performance and life cycle analysis. Results Eng..

[41] Zhang J., Zhang C., Guo S., Zhou L. (2012). Tool wear detection based on machine vision in end milling. Prod. Eng..

[42] Jiang F., Li J., Yan L., Sun J., Zhang S. (2010). Optimizing end-milling parameters for surface roughness under different cooling/lubrication conditions. Int. J. Adv. Manuf. Technol..

[43] Long X., Balachandran B. (2010). Stability of up-milling and down-milling operations with variable spindle speed. J. Vib. Control.

